# Fully bio-based wood adhesives from lignin and zein protein

**DOI:** 10.1039/d6gc02129h

**Published:** 2026-06-24

**Authors:** Ruslan Gryaznov, Fengyang Wang, Mahmoud Mazarji, Alberto J. Huertas-Alonso, Ievgen Pylypchuk, Mika H. Sipponen

**Affiliations:** a Department of Chemistry, Stockholm University SE-10691 Stockholm Sweden mika.sipponen@su.se; b Department of Chemistry, Wallenberg Wood Science Center, Stockholm University SE-10691 Stockholm Sweden

## Abstract

Manufacturing of wood-based panels predominantly relies on formaldehyde-containing adhesives, whose volatile organic compound emissions raise serious concerns about indoor air quality and human health. Although bio-based adhesives derived from proteins, polysaccharides, or lignin have been explored, achieving strong bonding performance while maintaining green, scalable, and practical processability remains challenging. In this study, we present a waterborne wood adhesive in which zein protein is dispersed within a viscous micellar lignin gel. Drawing inspiration from the interactions between proteins and polyphenols, we designed this system to be fully bio-based and free of cross-linkers. When birch veneers were hot-pressed with this adhesive, the resulting three- to five-layer plywood materials showed competitive performance compared with existing bio-based adhesive formulations. Meanwhile, this approach also delivers favorable green metrics, including an Eco-scale score of 94, supported by the absence of organic solvents, a minimal number of steps, and the waterborne nature of the formulation. Notably, the adhesive can be debonded in a 70% ethanol–water solution, allowing its recovery and reuse. The combined results indicate the potential of the lignin–protein system as a sustainable alternative to conventional petroleum-based commercial adhesives for indoor use.

## Abstract 2

A scalable lignin–zein adhesive that forms strong joints and can be selectively reversed on demand, enabling fully biobased plywood manufacturing and reuse for indoor applications.

## Graphical abstract

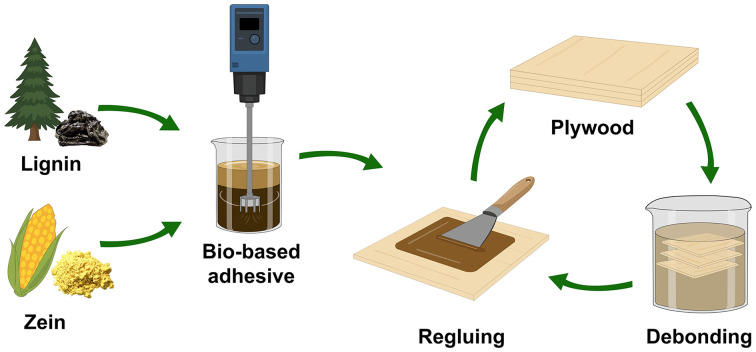

**Green foundation**

1. This work presents a fully bio-based, waterborne lignin–zein adhesive for plywood applications. Zein protein is dispersed within a lignin gel to form a continuous adhesive network without the need for synthetic cross-linkers or petroleum-derived resins, following green chemistry principles.

2. This adhesive formulation delivers competitive bonding performance together with favorable green metrics, including a high Eco-scale score, low *E*-factor and process mass intensity values, supported by its simple preparation, minimal processing steps and high material efficiency. The system exhibits debond-on-demand behavior, allowing bonded wood specimens to be separated in an ethanol–water solution, followed by recovery, reuse and reprocessing of the adhesive.

3. Future developments may focus on improving water resistance while preserving the debond-on-demand behavior, reusability and fully bio-based nature of the adhesive.

## Introduction

Petroleum-based adhesives such as urea–formaldehyde (UF), melamine–formaldehyde (MF), and phenol–formaldehyde (PF) systems continue to predominate in wood composites due to their high strength and rapid thermal curing.^1^ However, stricter environmental regulations and requirements to reduce formaldehyde emissions continue to drive demand for wood composites that meet environmental standards.^2,3^ In Europe, particularly in Sweden, wood-based panels are classified according to their formaldehyde emissions limit (*e.g.*, *E*_1_ ≤ 0.124 mg m^−3^), reflecting strict indoor air quality standards.^4^ There is thus a need for new bio-based adhesive formulations that simultaneously ensure low emission levels, sufficient adhesive strength, and compatibility with industrial applications.

To replace traditional petrochemical adhesives, a wide range of bio-based materials has been explored, including polysaccharides such as starch and hemicellulose, tannins, lignins, and plant-derived proteins.^5–11^ Natural biomacromolecules such as proteins and lignins are promising candidates for developing formaldehyde-free adhesives, considering their green and renewable source, high bonding strength, and potential biodegradability.^12,13^

Among readily available renewable biomass feedstocks, lignin has attracted increasing interest as an adhesive component, owing in part to its natural role as a binder in woody plant tissues. Because of its complex, mostly aromatic macromolecular structure, lignin can improve the mechanical properties of polymer matrices, making them more rigid, as well as resistant to heat and UV radiation.^14,15^ To overcome the poor and often incomplete solubility in water, aqueous dispersions of colloidal lignin particles can be prepared and used in a wide range of materials, including hair conditioners and bio-based adhesives.^16^ Lignin extracted in the presence of formaldehyde can be used as wood adhesives, demonstrating competitive performance in plywood applications.^17^ These studies highlight lignin as a promising component in the development of more environmentally friendly, sustainable adhesive systems that can fully replace petroleum-based analogs.

In recent years, increasing interest has been directed toward lignin–protein systems, in which interactions between functional groups govern their assembly and properties.^18^ Lignin can interact with amino acid residues of proteins through hydrogen bonds, van der Waals forces, π–π stacking, and cation–π interactions, forming a stable three-dimensional network.^19,20^ Despite the properties described above, the use of lignin–protein systems in fully bio-based adhesives remains limited. At the formulation level, lignin and proteins pose significant physicochemical challenges, as technical lignins are poorly water-soluble and structurally heterogeneous, while proteins are sensitive to pH and processing conditions, which affect their conformation and stability.^21^ As a result, blending often leads to heterogeneous systems or phase separation rather than a continuous network. Consequently, most of the described lignin–protein adhesive systems rely on chemical modification or external cross-linking agents to achieve strong adhesion.^22,23^ Therefore, the development of a simple, water-based adhesive system completely free of crosslinking agents remains challenging and a relatively unexplored area.

Among the available technical proteins, zein, a hydrophobic protein resulting from corn milling, has lately attracted interest. The low content of essential amino acids (in particular, lysine and tryptophan) makes zein an excellent bio-based candidate for developing sustainable materials as it does not compete with human feeding.^24,25^ Zein is used as a film former, a matrix for encapsulating bioactive substances, and as a basis for biodegradable packaging materials and adhesive systems.^26,27^ However, despite these advantages, zein's pronounced hydrophobicity hinders the formation of stable, homogeneous aqueous mixtures, limiting its use in fully water-based adhesive formulations.

This work presents a facile method for preparing a waterborne, fully bio-based adhesive that contains neither formaldehyde nor organic solvents. The adhesive is prepared by mixing lignin gel with zein, followed by adjusting the water content to the required solids content.^28^ The resulting composition demonstrates strong adhesion to wood and allows the production of three- and five-layer plywood under hot-pressing conditions. In addition to bonding performance, the system was evaluated for controlled debonding and adhesive reuse. In this work, reuse refers to the separation of bonded wood components in a 70% ethanol–water (w/w) solution under mild heating, followed by partial extraction, reconstitution and reuse of the adhesive material. This concept is relevant for end-of-life disassembly of indoor wood-based products and temporary wooden constructions, where controlled debonding may enable partial recovery and reuse of the adhesive material. It may also be useful in manufacturing scenarios where incorrectly bonded parts need to be debonded and the adhesive material to be recovered. Thus, the developed system represents a waterborne, bio-based adhesive platform with strong dry bonding performance and is suitable for debonding-on-demand applications.

## Results and discussion

### Waterborne lignin–zein adhesive formulation and curing behavior

**Fig. 1:**
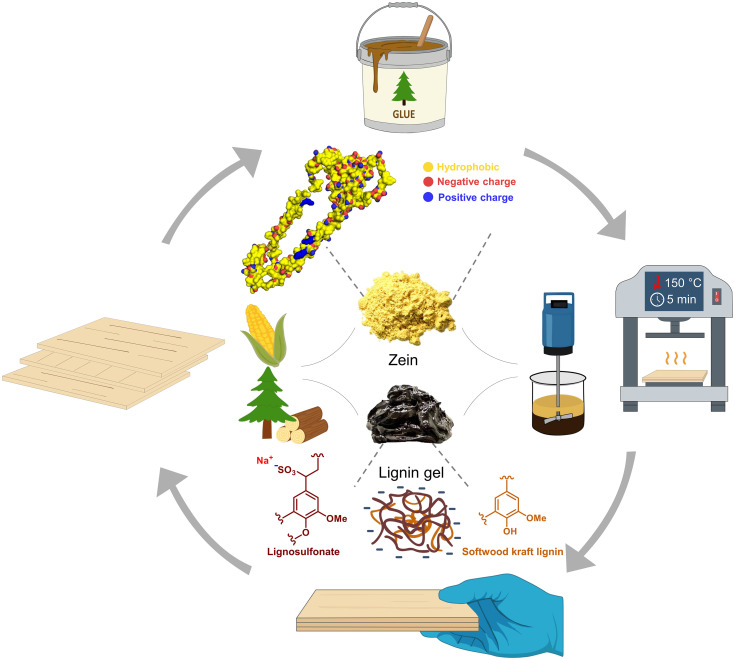
Preparation and application of lignin–zein protein adhesive in plywood manufacturing.

The goal of this work is to develop a bio-based adhesive aligned with the principles of green chemistry, avoiding the use of organic solvents or external cross-linking agents.^29^ The formulation is based on a lignin gel formed through dissolution of poorly water-soluble softwood kraft lignin under alkali conditions with sodium lignosulfonates, followed by pH neutralization.^28^ During the neutralization step, lignin self-organization leads to the formation of a stable colloidal micellar gel. This enables the production of the lignin gel without any organic solvents or energy-intensive evaporation steps, which is a key advantage of this method. To prepare adhesives, freshly made lignin gel was mixed with zein at high shear rates until a homogeneous dispersion was obtained (Fig. 1). Water was added to adjust the formulation to 42 wt% solids content, selected to balance adhesive loading, rheological properties, and processing stability during veneer application and hot-pressing.

To evaluate adhesive processability, the apparent viscosity of the lignin gel and lignin–zein formulations was measured as a function of shear rate (Fig. S1). The lignin gel showed shear-thinning behavior, with apparent viscosity decreasing as the shear rate increased. This behavior was retained after zein addition, indicating that the lignin–zein formulations remained suitable for spreading despite the incorporation of water-insoluble zein. The apparent viscosity varied with the lignin-zein ratio, and the formulation containing 30% lignin showed the highest viscosity at low shear rates. However, the lignin–zein adhesives remained spreadable and could be applied onto wood veneers. This spreadability is important for adhesive processing, as the material becomes easier to distribute under shear during application. Thus, the lignin gel acts as a water-based dispersing matrix that enables zein incorporation without compromising practical adhesive application. The entire formulation remains water-based and formaldehyde-free, with the advantage that external cross-linkers are not required.

**Fig. 2:**
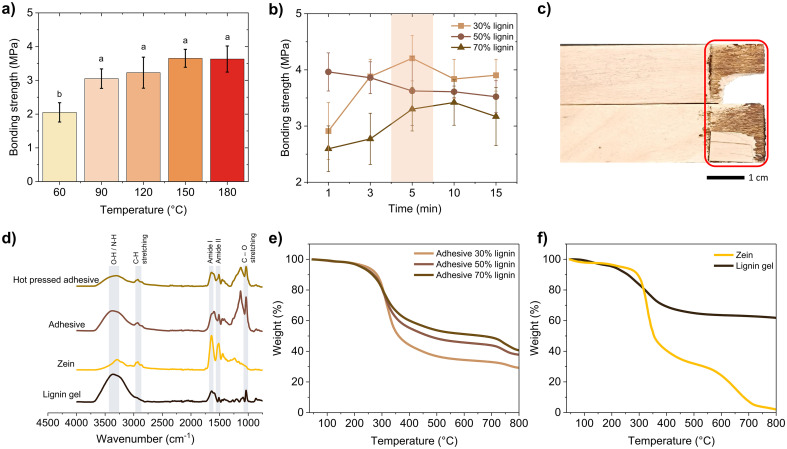
Multifaceted characterization of lignin–protein adhesives: (a) Optimization of the hot-pressing conditions for the adhesive (15 minutes curing at a temperature range from 60 to 150 °C). Different letters over the bars indicate significant differences between groups according to Tukey's *post-hoc* test following one-way ANOVA (*α* = 0.05). Groups sharing the same letter are not significantly different. (b) Adhesive strength kinetics for three different formulations (30, 50, 70% lignin content) at 150 °C from 1 to 15 minutes. (c) A photograph showing wood failure after tensile strength testing of a sample (10 minutes curing, 50% lignin adhesive formulation). (d) FTIR spectra for zein protein, lignin gel, adhesive before and after curing. (e) TGA analysis of different adhesive formulations (30, 50, 70% lignin content). (f) TGA analysis of zein and lignin gel samples.

To choose optimal operating conditions, a series of adhesive curing tests was conducted at temperatures ranging from 60 to 180 °C, at a constant pressure (1 MPa) and curing time (15 minutes). As shown in Fig. 2a, the bonding strength increased with curing temperature, giving average tensile shear strengths of 2.1 ± 0.3, 3.1 ± 0.3, 3.2 ± 0.5, 3.7 ± 0.3, and 3.6 ± 0.4 MPa at 60, 90, 120, 150, and 180 °C, respectively. Statistical analysis was further used to confirm the overall effect of curing temperature on bonding strength (one-way ANOVA, *p* < 0.05, *n* = 5), supporting the importance of thermal curing for adhesive performance. In the range from 60 to 150 °C, the increase in strength could be associated with dehydration and densification of the matrix formed by lignin gel and zein. Since increasing the temperature from 150 to 180 °C did not significantly improve bonding strength, 150 °C was selected as the practical reference temperature for subsequent time-optimization experiments. This choice balances processing efficiency with material integrity. It specifically minimizes unnecessary thermal exposure and mitigates the risk of wood degradation and delivers a robust high-strength curing point for evaluating shorter pressing times, rather than to define the lowest possible curing temperature. In accordance with the previously selected temperature of 150 °C, the dependence of the strength of a single-lap wooden joint as a function of the pressing time (1–15 minutes) was studied at the same pressure (1 MPa), preparation conditions, and different mass ratios of lignin gel and zein (30, 50, 70% of lignin content). For each formulation, five replicates were performed, and the results are shown in Fig. 2b. The following trend was observed for the strength of the adhesive bond: as time increased from 1 to 5 minutes, the strength increased, remained high at 10 minutes, and then followed by a slight decrease when the curing time reached 15 minutes. While comparing the bonding strength at a fixed curing time, it was found that an increase in protein content led to greater strength. It is noteworthy to mention that in the case of adhesive formulation with higher protein content, the nature of the break appears in the form of wood failure (Fig. 2c), confirming the formation of a strong interface and adhesive network in the joint. When the curing time was increased to 15 minutes, a slight decrease in strength appeared. This could be explained by over-drying of the adhesive joint and the formation of micro-cracks, as well as by over-compaction associated with joint brittleness. From this point of view, 5 minutes was selected as an optimum curing time for the subsequent samples. This duration provides maximum mechanical properties with minimum heat exposure, processing time, and energy consumption, resulting in short, efficient curing cycles in line with hot-pressing at an industrial scale.

To gain insights into structural changes, Fourier transform infrared spectroscopy (FTIR) was used to analyze individual components that compose the adhesive, zein and lignin gel, and adhesive itself before and after hot-pressing (Fig. 2d). The zein protein spectrum shows characteristic protein absorption bands. The broad band observed at 3300–3400 cm^−1^ corresponds to overlapping O–H and N–H stretching vibrations associated with hydroxyl groups and amide functional groups linked by hydrogen bonds. The bands at 1650 and 1540 cm^−1^ correspond to amide I (C=O stretching) and amide II (N–H bending and C–N stretching) vibrations, respectively, which are typical features of the secondary protein structure.^30^ Additional bands in the 1450–1400 cm^−1^ and 1230–1050 cm^−1^ correspond to C–H bending and C–N/C–O stretching of the protein backbone, respectively. The lignin gel exhibits characteristic bands of aromatic structures. A broad peak at 3400 cm^−1^ corresponds to the stretching vibrations of phenolic and aliphatic O–H groups. The band in the 1200–1000 cm^−1^ region refers to C–O stretching vibrations associated with phenolic groups.^31^ The presence of amide I and amide II bands, together with aromatic lignin bands, confirms that both lignin and protein phases are preserved in the adhesive composition. After hot-pressing at 150 °C, slight changes were observed, with the general positions of the bands remaining largely unchanged. This indicates that no new covalent chemical bonds were formed during the curing process. However, a broadening of the O–H/N–H band at 3300–3400 cm^−1^ suggests changes in hydrogen bonding interactions within the lignin–zein adhesive matrix. These observations support a curing process mainly associated with non-covalent interactions between lignin and zein, including hydrogen bonding, hydrophobic interactions between lignin aromatic domains and non-polar regions of the protein, and van der Waals interactions.

**Fig. 3:**
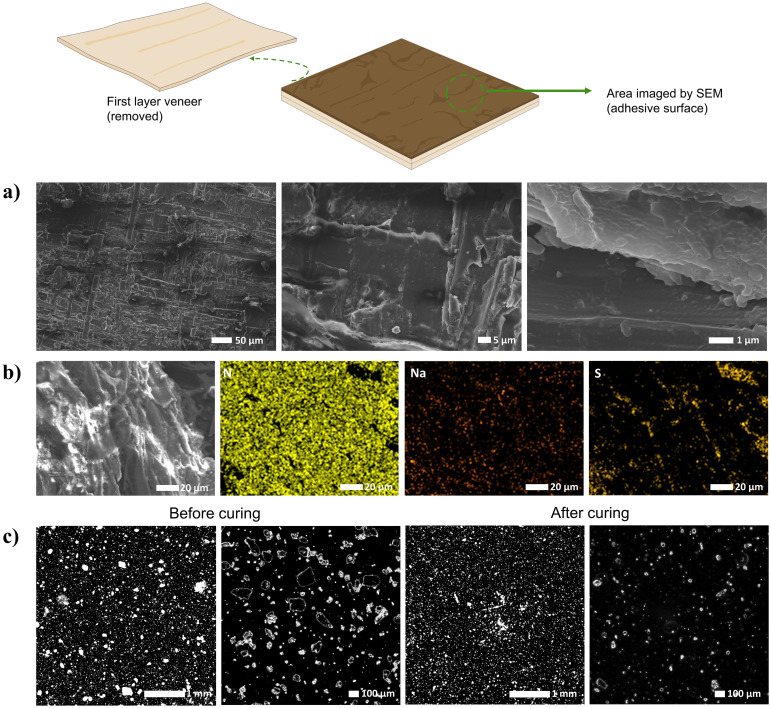
Morphological and elemental characterization of the adhesive: (a) SEM images of the adhesive surface (50% lignin, 150 °C, 15 minutes). (b) EDS analysis of the cured adhesive surface. (c) Optical microscopy images of the adhesive (50% lignin) before and after curing (5 minutes, 150 °C).

Thermogravimetric analysis (TGA) was performed under nitrogen atmosphere to evaluate the thermal degradation of pure zein and the lignin gel (Fig. 2f). All samples were pre-dried to minimize the influence of residual moisture. Pure zein demonstrated a sharp initial decomposition step between 280–380 °C, corresponding to the protein degradation range.^32^ In contrast, the lignin gel displayed a slower degradation over an applied temperature range, consistent with its complex aromatic structure and its well-known char-forming ability under inert conditions.^33^ The lignin–zein adhesive demonstrates intermediate thermal behavior with an identical decomposition region as for pure zein, but with a less sharp mass loss profile, and can be compared with adhesive formulations containing varying amounts of lignin (Fig. 2e). All samples showed a similar thermogravimetric behavior with a main decomposition step at a temperature range from 250 to 350 °C. As expected, an increase in lignin content led to a higher residual mass due to its char formation ability.^34^ Notably, no significant weight loss was observed near 150 °C, and the onset of the major decomposition step occurred at temperatures above 250 °C. This demonstrates that the hot-press curing occurred well below the thermal decomposition range of the adhesives. While the absence of the major mass loss at 150 °C indicates stability against thermal decomposition, one cannot exclude conformational rearrangement, denaturation, or aggregation of zein during hot-pressing. Heat-induced structural changes in zein have been reported previously, including changes in intermolecular interactions, protein configuration, and aggregate morphology after thermal treatment.^35^ In the present adhesive, zein is not present as an isolated protein phase but is incorporated into a lignin-rich water-based matrix, which acts as a dispersing medium under aqueous conditions. Therefore, any thermally induced zein restructuring would occur in the presence of lignin, where hydrogen bonding and restricted protein mobility may influence the final morphology of the cured adhesive. Such thermally induced restructuring may contribute to the consolidation of the lignin–zein adhesive matrix during curing. The morphology of the cured lignin–zein adhesive surface hot-pressed on a wood veneer (50% lignin, 150 °C, 15 minutes) was analyzed by scanning electron microscopy (SEM, Fig. 3a). SEM images reveal a continuous surface with no visible phase separation or macroscopic cracks/ruptures. This visual observation indicates effective densification of the adhesive layer under the chosen curing conditions. To evaluate the distribution of chemical elements across the adhesive surface, energy-dispersive X-ray spectroscopy (EDS) mapping was used (Fig. 3b). Dominant carbon (C) and oxygen (O) signals, corresponding to the organic nature of both zein and lignin, were observed across the whole scanned region. The nitrogen (N) signal, derived mainly from the protein fraction, was evenly distributed across the adhesive surface, indicating uniform incorporation of protein into the adhesive matrix. Further, sodium (Na) and sulfur (S), associated with salts in the lignin gel, were distributed across the sample. The absence of clear element-rich domains suggests that the cured adhesive surface remained uniform at the observed scale.

Optical microscopy was used to visualize the adhesive morphology in water before and after curing (Fig. 3c). The non-cured adhesive exhibits a dispersed microstructure, characterized by agglomerates of varying sizes distributed throughout the phase. The presence of large agglomerates is attributed to the salts in the lignin gel formulation, while some larger aggregates likely originated from the dispersed protein aggregates in the aqueous mixture. After curing at 150 °C, the morphology became more homogeneous, with a greater number of smaller particles and fewer large aggregates. This observation indicates thermally driven reorganization of the adhesive network, as evidenced by the mechanical properties observed in subsequent tensile strength tests.

### Mechanical performance of plywood and sawdust-based boards

**Fig. 4:**
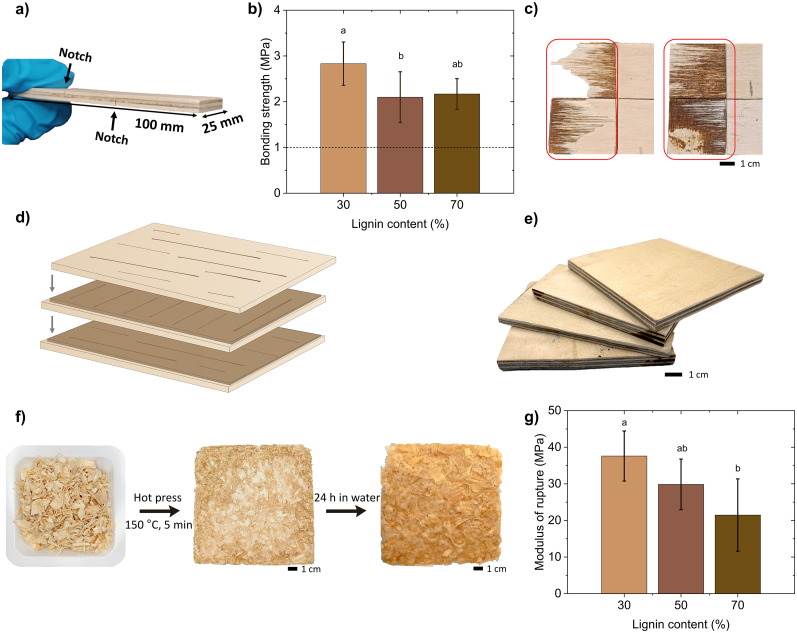
Engineering assessment for plywood and particleboard: (a) Plywood specimen with notches for mechanical testing according to EN-314 standard protocol. (b) Tensile strength of three-layer plywood hot pressed at 150 °C for 5 minutes, dashed line indicates the benchmark for dry plywood bonding. (c) A photograph showing wood failure after tensile strength testing of three-layer plywood samples (50% adhesive formulation). (d) Plywood orientation. (e) Images of three- and five-layer plywood. (f) Particleboard prepared from sawdust. (g) Modulus of rupture (MOR) of sawdust-based particleboards. Different letters over the bars in panels (b) and (g) indicate significant differences between groups according to Tukey's *post-hoc* test following one-way ANOVA (*α* = 0.05). Groups sharing the same letter are not significantly different.

To evaluate the adhesive's characteristics in a more practical context, plywood samples were prepared using optimized adhesive conditions. For the preparation of three-layer plywood, birch veneers wtih a 1.5 mm thickness were used. The moisture content of the veneers before gluing was 8%. The adhesive was applied with a dry spread rate (76 g m^−2^) over one side and evenly distributed by spatula. A three-layer plywood was assembled, with the middle veneer layer oriented with the fibers along the axis and the outer layers placed perpendicular, thereby achieving classic cross-layering to minimize warping and anisotropy (Fig. 4d). After hot-pressing at 150 °C under 1 MPa for 5 minutes, the samples were cooled and trimmed. The specimens for measuring strength were cut according to the EN-314-1 standard.^36^ Each specimen was 100 × 25 mm, with a notch on each side that intersected the entire first layer and half of the middle layer (Fig. 4a). The same was done on the other side, ensuring that the distance between the clamps during measurement was more than 50 mm. Before tensile measurement, each sample was conditioned for 24 hours at 23 °C and 50% relative humidity (RH). The measurements were performed under dry conditions, without water immersion pretreatment.

To clarify the role of the individual components, single-component control formulations were evaluated under the same water conditions. The lignin gel alone formed a spreadable paste but did not form a stable bonded joint after hot-pressing. The zein only control, in contrast, could not be processed into a homogeneous water-based adhesive paste due to its limited solubility, and instead formed an aggregated, poorly spreadable material (Fig. S2).

The average tensile shear strengths of the samples were 2.8 ± 0.5, 2.1 ± 0.6, and 2.2 ± 0.3 MPa for the adhesive formulations containing 30, 50, and 70% lignin, respectively (Fig. 4b and Fig. S3). For each measurement, six samples were tested (*n* = 6). One-way ANOVA showed that variation in the adhesive formulation had a significant effect on bonding strength (*p* = 0.028). Tukey *post-hoc* analysis showed that the bonding strength of 30% lignin formulation was statistically higher than the 50% lignin formulation (*p* = 0.038), while the other pairwise comparisons did not reach statistical significance at *p* < 0.05 level. The tensile strength measurements were accompanied by wood failure (Fig. 4c), in accordance with the adhesive's surface contribution from zein and the cohesive role of the lignin gel. The average shear strength values significantly exceeded 1.0 MPa, a commonly used reference threshold in plywood bonding assessments. The applicability of the adhesive for the preparation of multilayer plywood was further demonstrated by preparing five-layer plywood with a similar cross-layering orientation, meaning thateach neighboring layer is perpendicular to the previous one (Fig. 4e). The same pressing conditions were used as for the three-layer plywood manufacturing.

To demonstrate the adhesive's versatility beyond plywood, sawdust-based particleboards were prepared and quantitatively characterized (Fig. 4f). The boards were formed in a 160 × 160 × 4.3 mm mold. The same lignin–zein adhesive formulations used for plywood bonding were used for sawdust-based particleboard preparation. The stock adhesive formulation had a solids content of 42 wt% and was diluted with water before mixing with sawdust to improve adhesive distribution throughout the sawdust particle network. For each board, 89 g of sawdust was mixed with dry adhesive solids, corresponding to an adhesive loading of approximately 7.2 wt%, based on dry sawdust. The resulting water-diluted adhesive formulation had a solids content of 8 wt%. After forming, the boards were hot-pressed at 150 °C under 1 MPa for 5 minutes, using the same pressing conditions as for the plywood materials. Water absorption and thickness swelling were measured after 2, 6, 12, and 24 h of immersion in water. After 24 h immersion, the water absorption values were 152 ± 13%, 165 ± 22%, and 154 ± 16%, while the thickness swelling values were  98 ± 8%, 122 ± 15%, and 144 ± 8% for the adhesive formulations containing 30, 50, and 70% lignin, respectively (Table S1). Three-point bending tests were performed to evaluate the mechanical performance of the pressed boards. The modulus of rupture (MOR) values were 38 ± 7, 30.0 ± 7, and22 ± 10 MPa for the adhesive formulations containing 30, 50, and 70 lignin, respectively (Fig. 4g). These results demonstrate effective bonding of sawdust particles by the lignin–zein adhesive. Among the tested compositions, the 30% lignin formulation provided the best overall particleboard performance, combining the highest flexural strength with the lowest thickness swelling at all measured time points and comparable water absorption.

### Interfacial bond-line morphology and adhesive distribution

**Fig. 5:**
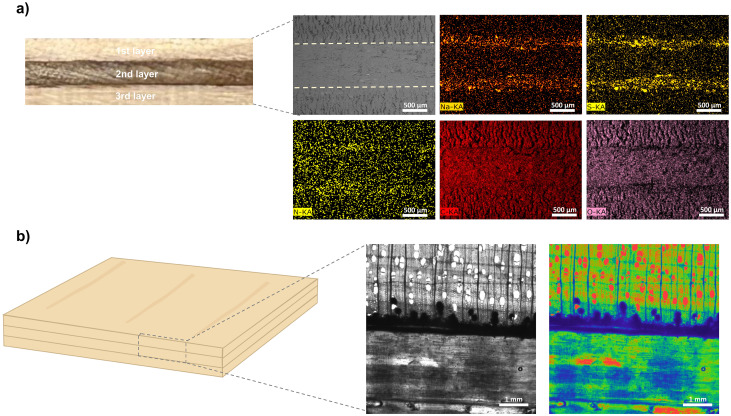
Microstructure analysis of the plywood adhesive joint (30% lignin adhesive formulation, 150 °C, 5 minutes): (a) SEM image of the wood-adhesive interface with EDS mapping, (b) Optical microscopy images of a microtome-cut plywood cross-section; the right image is color-coded for visual clarity.

To better understand the structure of the adhesive layer and its interactions with wood, optical and SEM images of plywood cross-sections were captured (Fig. S4). The cross-section of three-layer plywood, pressed at 150 °C under 1 MPa for 5 minutes, was characterized by SEM equipped with EDS mapping (Fig. 5a). For both SEM and EDS mapping, an accelerating voltage of 15 kV was used. The cross-sectional images showed the adhesive line as a continuous, dense layer between neighboring wood veneer layers, without visible microcracks and with uniform distribution along the entire length of the adhesive joint. This suggests that water evaporation during hot-pressing did not disrupt the adhesive layer. Although the formulation is water-based, the adhesive is a viscous lignin–zein paste that helps to maintain the adhesive components at the interface during heating. As water is gradually removed, the lignin–zein components become concentrated between the veneer layers, contributing to densification of the bond line. EDS mapping reveals the chemical structure of the narrow adhesive line between three layers of veneer, where S signals indicate the presence of lignosulfonates and Na signals originate from the corresponding salts in the lignin gel. Outside the joint, on the wood surface, the intensities of S and Na drop to background levels, consistent with their absence in the wood. The N signal, an identifier of zein protein due to its amino groups, was more widely distributed not only in the adhesive layer but also in the wood parts on both sides of the adhesive seam. This could be due to possible contribution from nitrogen-containing species in technical lignin and residual nitrogen-containing components naturally present in wood veneer.^37^ Signals from C and O dominated the entire surface of the specimen, without clear localization. These observations are consistent with the adhesive EDS analysis shown in Fig. 3b, confirming a homogeneous distribution of lignin and zein components within the adhesive formulation, without the formation of distinct segregated domains (Fig. S5 and S6).

Optical microscopy was used to analyze cross-sections of plywood and visualize the bonding morphology (Fig. 5b). The sections with 20 μm thickness were prepared with a microtome perpendicular to the adhesive interface. The optical images show an area with two glued veneers separated by a clear adhesive layer in the middle. As seen in the color-coded optical image, the lignin–zein-based adhesive formed a continuous bonding line at the interface, clearly distinguishable from the surrounding wood tissue. Near the interface, local penetration of the adhesive into the surface vessels and lumens was observed, although no overpenetration into the main wood structure was detected. The wood remains unchanged, with no large breaks or gaps, indicating a uniform distribution of the adhesive layer at this scale. Therefore, the observed rigid adhesive matrix, free of cracks or defects and partially penetrating the wood's surface layer, provides plywood with high strength and is consistent with the observed wood failure pattern shown in Fig. 4c.

### Debonding and adhesive recovery

**Fig. 6:**
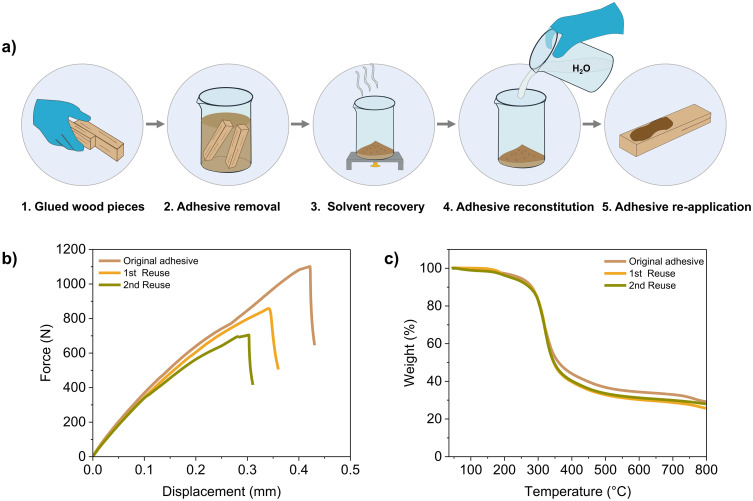
Reusing of lignin–protein adhesives: (a) Schematic illustration of reusing lignin–protein adhesives. (b) Force-displacement curves of glued wood samples after first and second reusing cycles (150 °C, 5 minutes hot-press curing, 30% lignin). (c) TGA analysis of the reused adhesive.

The increasing use of wood materials requires not only strong adhesive performance but also the ability to debond bonded components and recover the adhesive for reuse, enabling its return to the production cycle with minimal loss.^38^ Consequently, increasing attention is being focused on the development of debonding-on-demand adhesives that enable controlled disassembly upon exposure to external triggers scuh as solvents, heat, or pH changes.^39–41^ As shown in Fig. 6a, a recovery and reuse method for the lignin–protein adhesives was developed and tested. Single-lap specimens bonded by hot-pressing at 150 °C for 5 minutes were first immersed in 70% ethanol–water solution at 60 °C for 30 minutes (Step 2). A color change occurred in the solution after a few minutes, indicating the extraction of adhesive components from the bonded joint. The resulting solution was filtered to separate insoluble wood-derived residues, and then filtered solution was slowly evaporated at 40 °C (Step 3). In Step 4, the adhesive reconstitution was done by adding water to adjust the working solids content to 42%. In the final step, the reused adhesive was used to bond new single-lap wooden joints using the same pressing format, with a glued section area of 272 mm^2^. The recovery yield of adhesive was calculated from the dry mass of recovered adhesive relative to the dry mass of adhesive initially used in the bonded joints. The recovery yields were found to be 62% after the first recovery cycle and 70% after the second recovery cycle. These yields are consistent with the mild extraction conditions, which were selected to enable debonding and recovery without harsh chemical treatment or severe damage to the wood substrate. Under these conditions, part of the adhesive is expected to remain inside surface pores or attached to the wood cells after hot-pressing. Importantly, despite the partial material recovery, the recovered adhesive retained functional bonding ability. The strength of the glued specimens was evaluated by the maximum breaking force measured on the Instron Universal. For the original sample, before reusing, the maximum breaking force reached 1101 N (4.0 MPa shear strength). After the first and second reusing cycles, the maximum breaking force dropped to 858 N (3.1 MPa) and 705 N (2.6 MPa), respectively (Fig. 6b and Fig. S7). The gradual decrease in bond strength after each reusing cycle could be explained by several factors. During initial pressing, the adhesive partially penetrated the wood's surface pores and adhered to the walls. Therefore, this prevented its full recovery during our mild ethanol–water extraction method. Another factor could be that, despite filtration, a small fraction of soluble wood extractives passed the recovery stage and ended up in the dry recovered adhesive, reducing both the adhesive's spreadability within the pores and its mechanical strength. In addition, possible irreversible thermal restructuring of zein during repeated hot-pressed cycles may also contribute to the decrease in bonding strength. As discussed above, zein may undergo conformational rearrangement, denaturation, and aggregation at the hot-pressing temperature. While such heat-induced restructuring may contribute to adhesive consolidation during curing, it may also reduce the ability of the recovered adhesive to fully redisperse and reform the same adhesive matrix in subsequent reuse cycles.

Thermogravimetric analysis (TGA) was performed to evaluate the thermal stability of the adhesives after each reusing step, compared with the original adhesive formulation containing 30% lignin (Fig. 6c). All samples exhibit similar thermogravimetric behavior, with a main decomposition step in the temperature range of 250–350 °C. The first and second reused samples showed nearly overlapping thermograms, indicating that the mild recovery procedure did not cause substantial changes in the thermal degradation profile of the adhesive. These results demonstrate the possibility of disassembling the adhesive joint and further functional reuse without significant degradation, which is essential for the huge sector of temporary wooden constructions.

### Assessment of chemical hazards and green metrics

Despite significant progress in developing bio-based wood adhesives, finding environmentally sustainable formulations that can compete with traditional petrochemical alternatives remains a major issue. A previous review paper has highlighted broad advances in lignin-, tannin-, protein-, and other biomass-derived adhesive systems.^42^ To compare the present adhesive with relevant lignin-based wood adhesive systems, we considered representative examples including lignin-glyoxal resins, uncondensed lignin adhesives, oxidized or demethylated lignin-soy protein adhesives, lignin-glycerol diglycidyl ether systems, and epoxidized lignin formulations.^3,17,22,23,43,44^ However, many of these systems still involve organic solvents, chemically modified lignin, epoxy-type chemistry, reducing agents, or additional reactive cross-linking components. In, the adhesive developed in this work is fully water-based, avoiding the use of organic solvents and cross-linking agents classified as carcinogenic, mutagenic, or toxic to reproduction (CMR). This simple formulation strategy leads to high green performance metrics.

The incorporation of lignin as a renewable component of materials is a recognized green strategy, highlighting the potential of lignin-containing binders to replace petrochemical systems.^8^ In this work, zein was dispersed in an aqueous lignin gel without the use of organic solvents or external cross-linking agents. The environmental profile of the adhesive preparation was evaluated using *E*-factor, process mass intensity (PMI), process mass productivity (PMP), and Eco-scale.^45^ The *E*-factor is defined as the mass of waste generated per mass of product, while PMI is defined as the total mass of input materials required to obtain one unit mass of product. In this work, both metrics were calculated using dry adhesive solids as the product basis, since water acts as the processing medium and is removed during hot-pressing. For the adhesive formulation containing 50% lignin, the *E*-factor was 1.34, and the PMI was 2.34 (Table S2). A PMI of 2.34 means that 2.34 kg of total input material is required to obtain 1 kg of dry adhesive solids. PMP expresses the same mass-efficiency relationship in percentage form and was calculated as the inverse of PMI: PMP = 1/PMI × 100%. Thus, the PMI value of 2.34 corresponds to a PMP of 42.6%. Since the adhesive is prepared as a waterborne formulation, the non-product mass in these calculations is associated with water removal during hot-pressing rather than hazardous solvents. Eco-scale was used as a complementary penalty-based assessment of the adhesive preparation (Table S3). The score was calculated by starting from an ideal value of 100 and subtracting penalty points for yield, reagent cost, safety, temperature/time, or technical setup. In this process, no penalty was assigned for safety or workup/purification, as the formulation uses low-hazard bio-based components and water as the processing medium. A penalty of 3 points was assigned for the technical setup because hot-pressing requires pressure equipment, and an additional penalty of 3 points was assigned for temperature/time requirements, accounting for overnight stirring during lignin gel preparation. Therefore, the total penalty was 6 points, giving an Eco-scale score of 94. Together, these values indicate a favorable process profile for the lignin–zein adhesive preparation, combining reasonable material efficiency with a waterborne, solvent-free formulation and a high Eco-scale score.

**Fig. 7:**
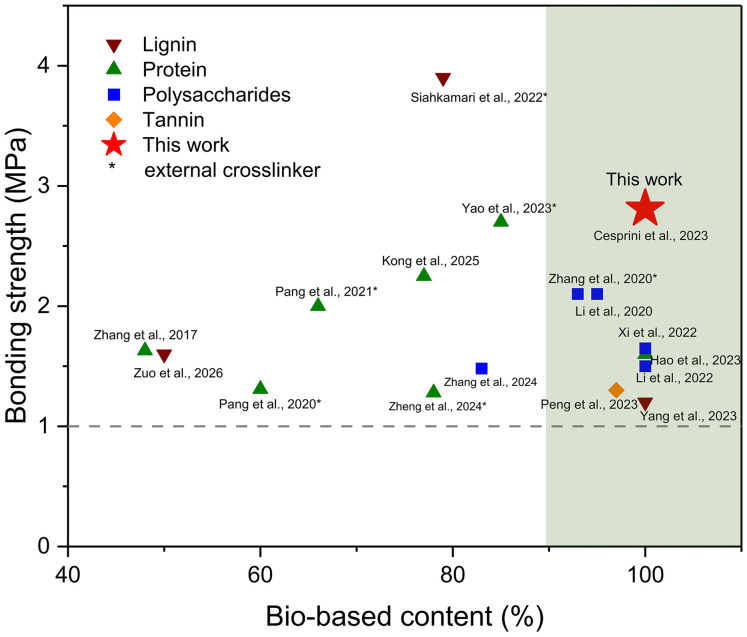
Comparison of dry bonding strength to bio-based content for three-layer plywood.^3,17,46–60^ The values used in this graph can be found in Table S4.

As shown in Fig. 7, the strength of fully bio-based adhesives is generally limited, whereas higher performance is often associated with chemical modification or partial substitution with non-bio-based components. This work provides competitive bond strength with 100% bio-based content in the absence of external cross-linking agents. It is particularly impressive, as it is achieved in the fully water-based formulation, indicating the potential to overcome the traditional limitations of bio-based adhesive systems and paving the way for the development of environmentally sustainable and highly effective alternatives for wood bonding.

## Conclusions

In this work, we developed a waterborne bio-based adhesive based on zein protein and lignin gel, without the use of formaldehyde, organic solvents, or external cross-linking agents. The adhesive system was systematically investigated in terms of processability, dry bonding performance, thermal behavior, particleboard applicability, green metrics, and reusability. Single-component control experiments further showed that the combined lignin–zein formulation provides a better balance of water-based processability and bonding performance than either component alone. The adhesive demonstrated strong dry bonding performance in plywood, with the formulation containing 30% lignin yielding the highest average tensile shear strength among the tested compositions. Microscopy and EDS analysis revealed the formation of a continuous adhesive line at the wood interface without visible macroscopic defects or distinct segregated domains. Beyond plywood, the adhesive was also applied to sawdust-based particleboards, where water absorption, thickness swelling, and three-point bending measurements showed composition-dependent performance. Among the tested particleboard formulations, the 30% lignin adhesive showed the best overall performance, combining the highest flexural strength with the lowest thickness swelling. The adhesive demonstrated potential for controlled debonding and reuse by extracting the adhesive material with a 70% ethanol–water solution under mild conditions, followed by reprocessing. Thermal analysis of the recycled adhesive indicated that the main thermal characteristics were retained after multiple reuse cycles, confirming that the adhesive system's structure was preserved. Overall, the results show that lignin and protein-based compositions can form effective bio-based adhesives without requiring external chemical cross-linking agents. The combination of high adhesive strength, practical processability, reusability, and favorable green metrics highlights the potential of lignin–protein-based systems as an alternative to traditional synthetic wood adhesives for indoor applications.

## Experimental

### Materials and methods

Sodium lignosulfonate (DS10, Domsjö, Sweden), softwood kraft lignin (SKL, BioPiva 100, UPM, Finland), zein (22–24 kDa, Sigma Aldrich), sulfuric acid (VWR, Sweden), sodium hydroxide (VWR, Sweden), wood veneers (1.5 mm thickness, Koskisen, Finland), wood specimens (1.5 mm thickness, Flying Tiger, Sweden), wood chips (Vitakraft, Bauhaus, Sweden). Detailed characterization data for the lignin components are provided in Table S5.

### Protein structure visualization

The zein protein structure shown in Fig. 1 was obtained from the AlphaFold Protein Structure Database and visualized using Open-Source PyMOL (The PyMOL Molecular Graphics System, Version 4.6, Schrödinger, LLC).^61,62^ The structure was used for schematic visualization of the zein component in the adhesive formulation.

### Colloidal lignin gel preparation

The colloidal lignin gel was prepared according to our earlier work.^28^ Briefly, sodium lignosulfonate (510 g, dry) and softwood kraft lignin (102 g, dry) were dissolved in a 5 : 1 ratio in 746 mL of 2 M sodium hydroxide solution, and the mixture was stirred overnight. Then, 144 mL of water was added at pH ∼ 13.5. Finally, 175 mL of 2 M sulfuric acid solution was added dropwise to obtain a lignin gel with a pH of ∼5.6. The obtained lignin gel solids content was ∼41%.

### Adhesive preparation

Different lignin-protein adhesive formulations were prepared with lignin contents of 30, 50, and 70%. The 1 : 1 ratio lignin–protein adhesive was prepared using 26.3 g of lignin gel dispersed in water (12.7 g), followed by the addition of 12.3 g zein to the colloidal solution. The mixture was homogenized by IKA T-25 Ultra-Turrax (IKA, Germany) for 3 minutes at 15 000 rpm. Prepared adhesives were stored at room temperature. The obtained adhesive solids content was ∼42%.

### Bonding strength sample preparation

50 mg of adhesive was applied to 272 square mm^2^ (dry ∼76 g m^−2^) on each wood specimens and cured for 1 to 15 minutes using a Fontijne LabTop Hot Press at 150 °C under 1 MPa pressure. All the samples were conditioned for 24 hours in a conditioning room (23.1 °C, 50% RH) after pressing.

### Plywood preparation

To prepare three-layer plywood, three layers of veneers (100 × 100 × 1 mm) were glued, adding 1.8 g of adhesive per layer (dry ∼76 g m^−2^) with a cross-layer orientation; each veneer grain is perpendicular to its neighboring layer. Glued veneers were cured in a Hot Press (Fontijne LabTop) at 150 °C for 5 minutes. All samples were conditioned for 24 hours (23.1 °C, 50% RH) prior to tensile strength measurements.

### Sawdust-based particleboard preparation

For particleboard preparation, the same stock lignin–zein adhesive formulation used for plywood gluing was diluted with water before mixing with sawdust. For each board, 89 g of sawdust was combined with 6.4 g of dry adhesive solids, giving a dry adhesive loading of 7.2 wt% based on sawdust. After dilution, the adhesive formulation used for sawdust mixing had 8 wt% solids content. The diluted adhesive was added gradually to the sawdust under intensive mixing until the particles were evenly moistened. The mixture was placed into a 160 × 160 × 4.3 mm mold and hot-pressed using a Fontijne LabTop Hot Press at 150 °C under 1 MPa for 5 minutes. The obtained boards were conditioned for 24 h at 23.1 °C and 50% RH before testing.

### Green metrics calculation

Green metrics were calculated for the adhesive formulations, following green chemistry metric definitions.^45^ The *E*-factor, process mass intensity (PMI), process mass productivity (PMP), and Eco-scale were calculated as follows: *E*-factor = mass of waste/mass of product; PMI = total mass used in process/mass of product; PMP (%) = (1/PMI) × 100; Eco-scale = 100 − total penalty points. Waste refers to the non-product material generated under the selected calculation boundary. For the Eco-scale assessment, penalty points were assigned based on yield, reagent cost, safety, technical setup, temperature/time, and workup/purification.

### Characterization methods

#### Rheological measurements

Rheological measurements were performed using a NETZSCH Kinexus rheometer equipped with a CP4/40 geometry at room temperature. The apparent viscosity of the lignin gel and lignin–zein adhesive formulations was measured as a function of shear rate.

#### Mechanical test

Mechanical strength measurements were performed by using an Instron 5960 series tensile tester (Instron, USA) equipped with a 10 kN load cell and connected to Bluehill software. The test was performed with a pulling speed of 2 kN min^−1^. The tensile shear strength was calculated as the maximum force divided by the bonded area. The flexural strength of particleboard specimens was measured by three-point bending using an Instron Universal testing machine equipped with 1 kN load cell. Rectangular specimens were cut from the pressed boards (160 × 20 × 4.3 mm), and the actual width and thickness of each specimen were measured before testing. The support span was set to 80 mm, and the crosshead speed was 2 mm min^−1^. The modulus of rupture (MOR) was calculated as MOR = 3*F*_max_*L*/2*bd*^2^, where *F* is the maximum force, *L* is the support span, *b* is the specimen width, and *d* is the specimen thickness.

#### Water absorption and thickness swelling

Water absorption and thickness swelling of particleboards were measured after immersion in water for 2, 6, 12, and 24 h. Specimens with dimensions of 50 × 50 × 4.3 mm were used for the measurements. Before immersion, the initial mass and thickness of each specimen were measured. After each immersion time, the specimens were removed from water, gently wiped to remove surface water, weighed, and measured for thickness. Thickness was measured at five points, and the average value was used for the calculation. For each adhesive formulation, three specimens were tested (*n* = 3). Water absorption and thickness swelling were calculated as follows: WA (%) = ((*m*_t_ − *m*_0_)/*m*_0_) × 100, TS (%) = ((*t*_t_ − *t*_0_)/*t*_0_) × 100, where *m*_0_ and *t*_0_ are the initial mass and thickness, and *m*_t_ and *t*_t_ are the mass and thickness after immersion time.

#### Fourier transform infrared spectroscopy

Fourier transform infrared spectra were recorded by using a Varian 610-IR FTIR Spectrometer in attenuated total reflection (ATR) mode in the range of 400–4000 cm^−1^ over 32 scans.

#### Optical microscopy analysis

Optical images were captured using a Nikon ECLIPSE Ti2 inverted microscope. Analysis of the adhesive before and after heat treatment was performed on 1.0 wt% diluted samples on the microscope slides, using 4× to 10× objective lenses. To evaluate adhesive penetration into the wood pores, cross-sections of plywood samples were prepared using a microtome to yield 20 μm-thick sections. The obtained images were color-enhanced by NIS-Elements software to improve the visualization of the adhesive bond.

#### Scanning electron microscopy

The morphology of the adhesive surface was studied by using JEOL JSM-IT800 scanning electron microscope equipped with an EDS detector at an accelerating voltage of 10–15 kV. Cross-sectional images of the plywood sample were captured by a Hitachi TM-3000 scanning electron microscope equipped with a Bruker Quantax 70 EDS system at an accelerating voltage of 15 kV for EDS mapping.

#### Thermal gravimetry analysis

To evaluate thermal stability, thermogravimetric analysis was performed using a PerkinElmer TGA 7 instrument. Samples were heated from 25 to 800 °C at a heating rate of 10 °C min^−1^ in a nitrogen atmosphere at a flow rate of 20 mL min^−1^.

## Author contributions

R. G. and M. H. S. conceived the idea and designed the experiments. I. P. and M. H. S. supervised the project. R. G., F. W., and M. M. performed the experiments. F. W., M. M., A. J. H., I. P., and M. H. S provided constructive suggestions. R. G., F. W., M. M., A. J. H., I. P. and M. H. S. contributed to data preparation, analysis and manuscript drafting with input from all authors. M. H. S. provided resources for this study. All authors discussed and revised the paper.

## Conflicts of interest

There are no conflicts to declare.

## Supplementary Material

GC-028-D6GC02129H-s001

## Data Availability

The source data supporting the findings of this study are openly available at Zenodo: https://doi.org/10.5281/zenodo.19478414. Supplementary information is available. See DOI: https://doi.org/10.1039/d6gc02129h.
