# Supplementary material for: Fully bio-based wood adhesives from lignin and zein protein
Source: Green Chem. 2026 Jun 24. Online ahead of print. doi: 10.1039/d6gc02129h (PMC13343468; doi:10.1039/d6gc02129h)
Supplement: GC-OLF-D6GC02129H-s001 [file GC-OLF-D6GC02129H-s001.pdf]

This journal is © The Royal Society of Chemistry 2026

# Supplementary information

## Fully bio-based wood adhesives from lignin and zein protein

*Ruslan Gryaznov[1,2], Fengyang Wang[1], Mahmoud Mazarji[1], Alberto J. Huertas-Alonso[1], Ievgen Pylypchuk[1], Mika H. Sipponen*[1,2]*

*[1]Department of Chemistry, Stockholm University, SE-10691, Stockholm, Sweden*

*[2]Deparment of Chemistry, Wallenberg Wood Science Center, Stockholm University, SE-10691, Stockholm, Sweden*

**Corresponding to mika.sipponen@su.se*

This PDF contains:
Figure S1-S7
Table S1-S5
Supplementary references

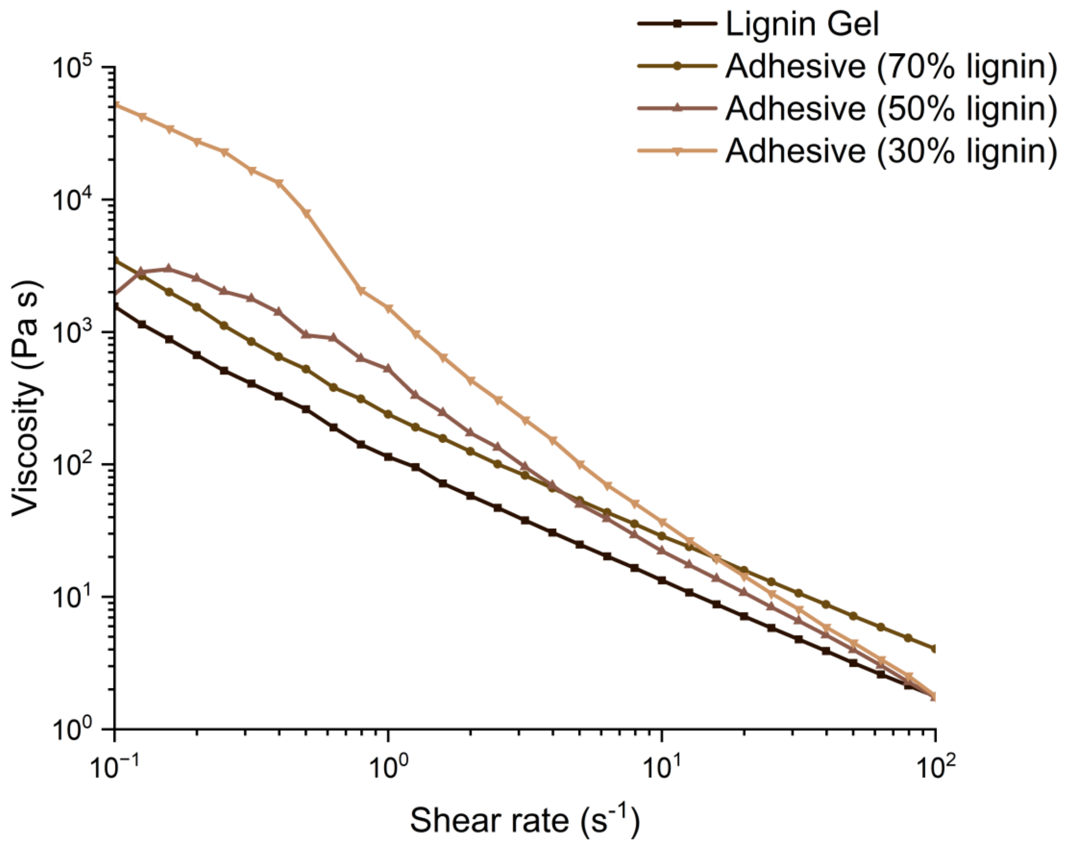


**Figure S1**. Viscosity of lignin gel and lignin-zein adhesive formulations as a function of shear rate.

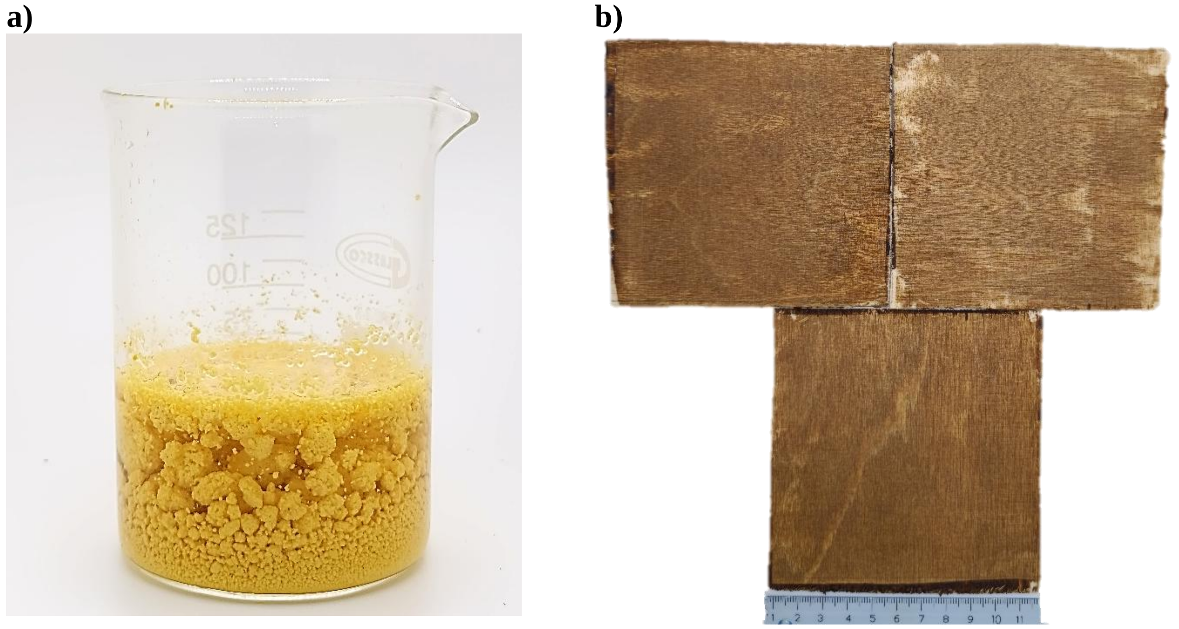


**Figure S2.** Control experiments for individual adhesive components. **a)** Zein dispersed in water showing inhomogeneous aggregation and lack of processable paste formation **b)** Lignin gel only plywood sample after hot pressing, showing delamination and insufficient bonding.

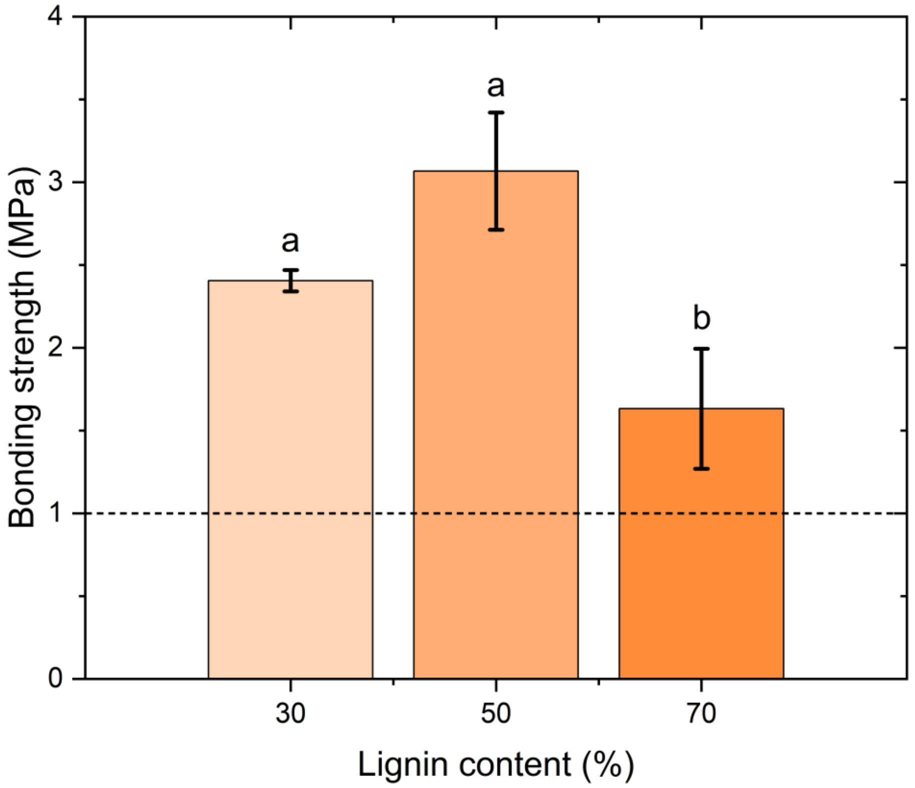


**Figure S3**. Bonding strength for three-layer plywood cured for 15 minutes at 150 °C. Different letters over the bars indicate significant differences between groups according to Tukey´s post-hoc test following one-way ANOVA (α = 0.05). Groups sharing the same letter are not significantly different.

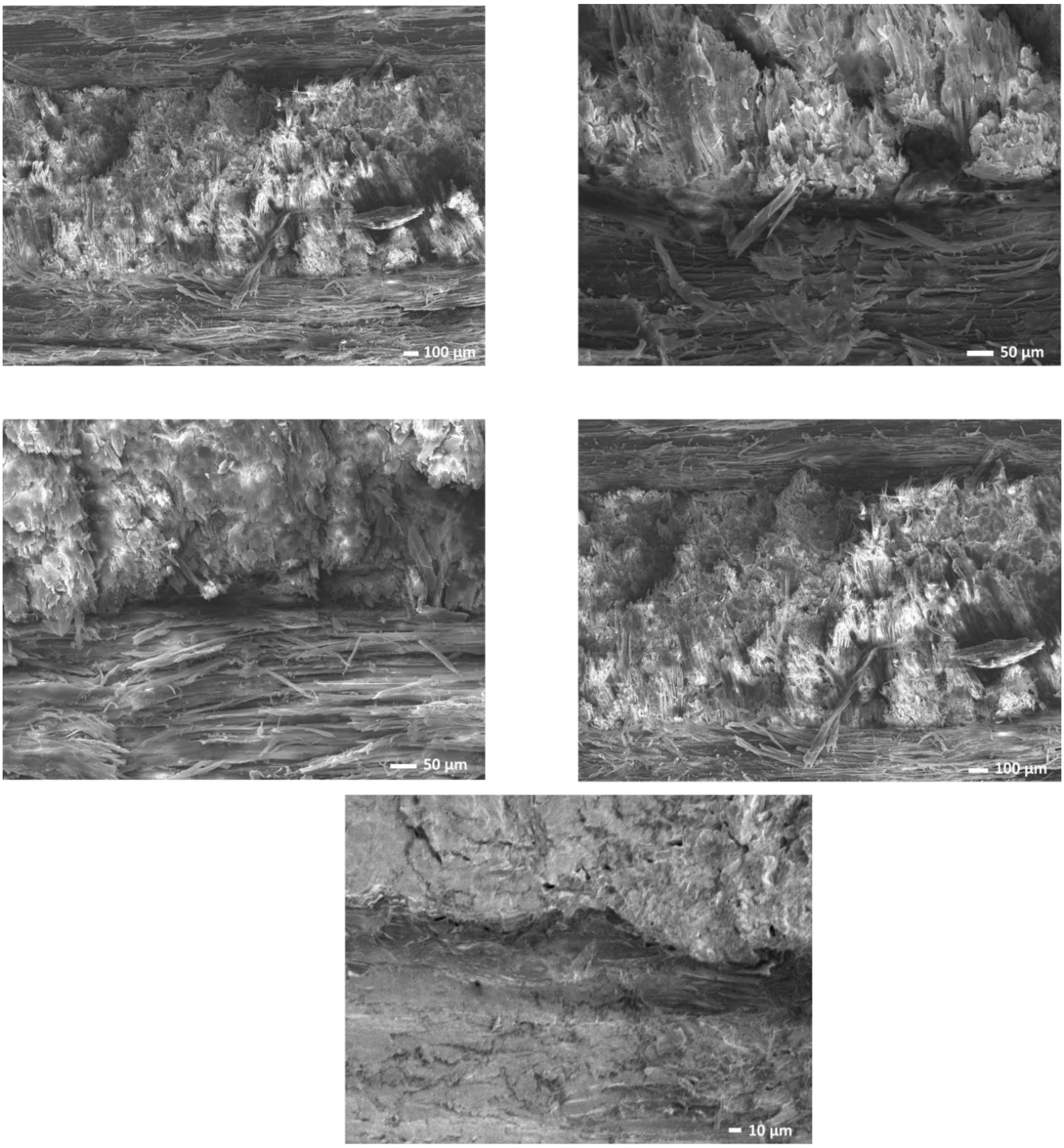


**Figure S4.** SEM images of plywood cross-section (30% lignin, 150°C, 5 minutes). Scale bars, from left to right: 100 µm, 50 µm (top row); 50 µm, 100 µm (middle row), 10 µm (bottom row).

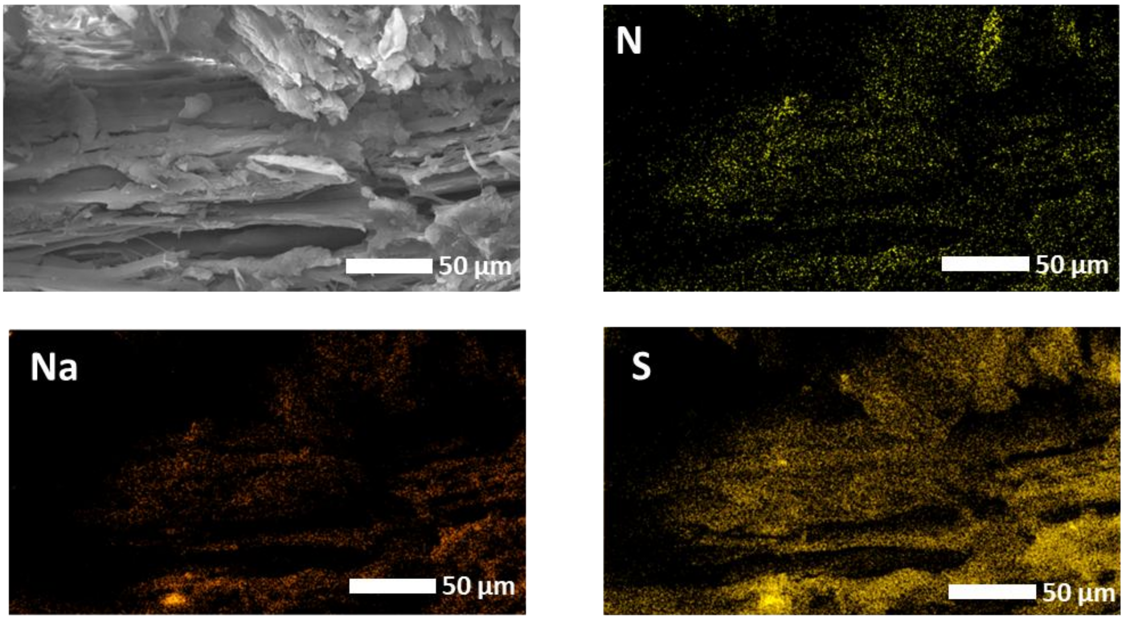


| Element | Line | Mass% | Atom% |
|---|---|---|---|
| C | K | 42.65±0.29 | 50.14±0.11 |
| N | K | 6.82±0.39 | 6.87±0.13 |
| O | K | 45.88±0.40 | 40.49±0.11 |
| Na | K | 2.78±0.05 | 1.71±0.01 |
| S | K | 1.23±0.02 | 0.54±0.00 |
| Cl | K | 0.33±0.01 | 0.13±0.00 |
| K | K | 0.32±0.01 | 0.11±0.00 |
| Pt | M | nd | nd |
| Total | | 100.00 | 100.00 |
| Map_006_wholespectrum | | | Fitting ratio 0.1528 |

**Figure S5.** EDS analysis of plywood cross-section (30% lignin formulation, 150 °C, 5 minutes).

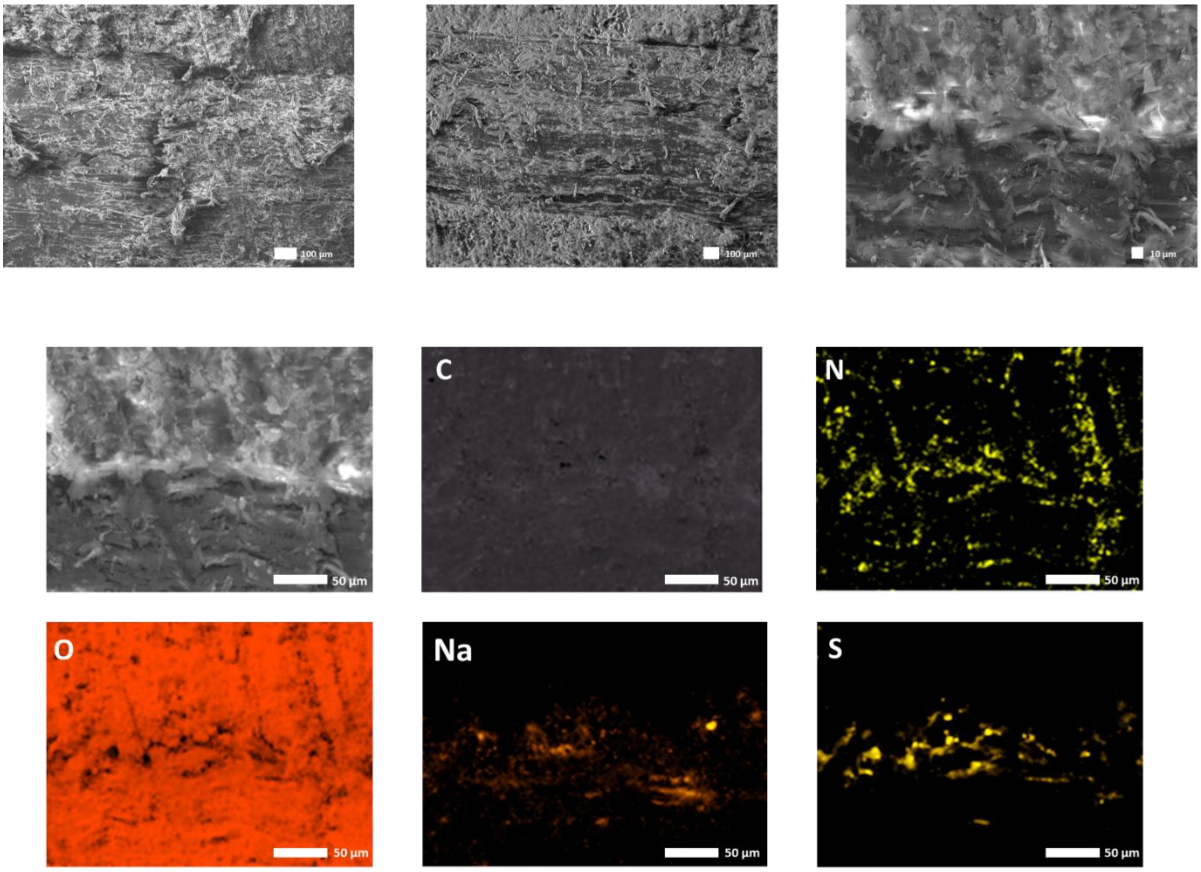


**Figure S6.** SEM and EDS analysis of plywood cross-section (50% lignin formulation, 150 °C, 15 minutes). Scale bars, from left to right: 100 µm, 100 µm, 10 µm (top row); 50 µm (other images).

**Table S1**. Water absorption and thickness swelling of sawdust-based particleboards. Different letters over the bars indicate significant differences between groups according to Tukey´s post-hoc test following one-way ANOVA (α = 0.05). Groups sharing the same letter are not significantly different.

| Formulation | WA 2 h (%) | WA 6 h (%) | WA 12 h (%) | WA 24 h (%) | TS 2 h (%) | TS 6 h (%) | TS 12 h (%) | TS 24 h (%) |
|---|---|---|---|---|---|---|---|---|
| **Adhesive 70% lignin** | 130.9 ± 19.3 [a] | 171.5 ± 28.0 [a] | 173.1 ± 26.7 [a] | 153.9 ± 15.8* [a] | 100.2 ± 15.5 [a] | 116.5 ± 12.8 [a] | 136.8 ± 9.8 [a] | 144.2 ± 7.7* [a] |
| **Adhesive 50% lignin** | 122.1 ± 411.8 [a] | 143.7 ± 15.2 [ab] | 156.2 ± 15.1 [a] | 165.2 ± 22.0 [a] | 81.1 ± 3.5 [ab] | 97.4 ± 11.4 [ab] | 113.1± 19.9 [ab] | 121.8 ± 1 5.0 [ab] |
| **Adhesive 30% lignin** | 112.2 ± 13.2 [a] | 117.3 ± 11.1 [b] | 136.3 ± 15.7 [a] | 151.9 ± 12.9 [a] | 72.5 ± 5.8 [b] | 81.6 ± 3.3 [b] | 90.3 ± 6.3 [b] | 97.9 ± 7.5 [b] |

* Partial material loss was observed for the 70% lignin formulation after 24 h immersion, which may affect the accuracy of the mass-based water absorption value.

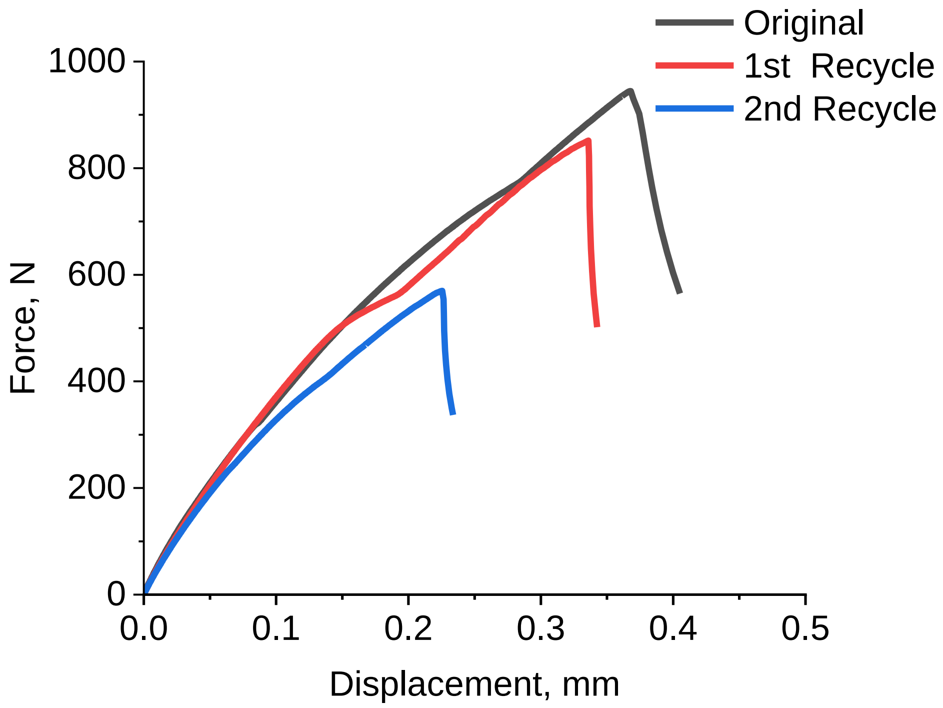


**Figure S7.** Tensile strength measurement for adhesive before and after 2 reusing cycles (50% lignin formulation, 5 minutes curing).

**Table S2**. Input masses used for green metrics calculation of the lignin-zein adhesive formulation containing 50% lignin.

| Component | Mass (g) | Dry content (wt%) | Dry solids (g) | Water mass (g) |
|---|---|---|---|---|
| **Lignin gel** | 52.50 | 41 | 21.53 | 30.97 |
| **Zein** | 22.00 | 96 | 21.12 | 0.88 |
| **Added water** | 25.50 | - | - | 25.50 |
| **Total input mass** | 100.00 | - | 42.65 | 57.35 |

**E-factor** is defined as the mass of waste generated per mass of desired product:

E-factor = mass of waste / mass of product = 57.35 / 42.65 = 1.34

In this work, the waste mass corresponds to the total water mass in the formulation, including water present in the lignin gel, residual moisture in zein and added water used for formulation adjustment since water is evaporated during hot pressing,

**Process mass intensity (PMI)** is defined as the total mass used in the process required to obtain one unit mass of product:

PMI = Total mass used in the process / mass of product = 100 / 42.65 = 2.34

**Process mass productivity (PMP)** expresses PMI as a percentage value:

PMP (%) = 1 / PMI × 100 = 1 / 2.34 × 100 = 42.6%

**Table S3**. Penalty points used for Eco-scale calculations of the lignin-zein adhesive.

| Parameter | Description | Penalty points |
|---|---|---|
| **Yields** | No penalty assigned | 0 |
| **Price of components** | No penalty assigned | 0 |
| **Safety** | No penalty assigned | 0 |
| **Technical setup** | Hot pressing / pressure equipment | 3 |
| **Temperature/time** | Overnight stirring during lignin gel preparation and hot pressing | 3 |
| **Workup and purification** | No penalty assigned | 0 |
| **Total penalty points** | - | 6 |

Eco-scale was calculated by subtracting penalty points from an ideal score of 100.

Eco-scale = 100 – total penalty points = 100 – 6 = 94

**Table S4**. Comparison of Dry Bonding strength and bio-based content for three-layer plywood adhesives.

| Material | Dry bonding strength (MPa) | Bio-based content, % | Ref |
|---|---|---|---|
| Uncondensed lignins | 1.2 | 100 | [1] |
| Lignin - glyoxal | 3.9 | 79 | [2] |
| Soy protein - acrylic acid | 2 | 66 | [3] |
| Soy protein - chitosan - $CaCO_3$ | 2.25 | 77 | [4] |
| Soy protein - epoxy | 1.31 | 60 | [5] |
| Cottonseed protein - Isophorone diisocyanate | 2.7 | 85 | [6] |
| Glucose - urea | 1.48 | 83 | [7] |
| Soy protein - citric acid | 1.6 | 100 | [8] |
| Tannin - furanic - silk | 2.8 | 100 | [9] |
| Soy protein - lignin - based resin | 1.63 | 48 | [10] |
| Enzymatic hydrolysis of soy protein | 1.28 | 78 | [11] |
| Chitosan - starch | 2.1 | 95 | [12] |
| Chitosan with monosaccharide | 1.65 | 100 | [13] |
| Glucose and citric acid | 1.5 | 100 | [14] |
| Tannin - polyamide | 1.3 | 97 | [15] |
| Lignin - formaldehyde | 1.6 | 50 | [16] |
| Soy bean polysaccharide - epoxy | 2.1 | 93 | [17] |
| This work | 2.8 | 100 | |

**Table S5**. Characterization data for the lignin components used for lignin gel preparation. Data adapted from our previous work.[18]

| Lignin type | $M_w$, Da | Ph-OH, mmol/g | Aliph-OH, mmol/g | COOH, mmol/g | Dispersity, Đ (Mw/Mn) | Klason Lignin (Acid insoluble lignin) % | Ref |
|---|---|---|---|---|---|---|---|
| SKL | 5250 | 4.21 | 1.93 | 0.56 | 4.4 | 92 | [19] |
| Lignosulfonate DS10 | 10482 | 0.83 | 3.27 | 0.74 | 5.2 | 96 | [20] |

## References:


1. Yang, G., Gong, Z., Luo, X., Chen, L. & Shuai, L. Bonding wood with uncondensed lignins as adhesives. *Nature* **621**, 511–515 (2023).
2. Siahkamari, M., Emmanuel, S., Hodge, D. B. & Nejad, M. Lignin-Glyoxal: A Fully Biobased Formaldehyde-Free Wood Adhesive for Interior Engineered Wood Products. *ACS Sustainable Chem. Eng.* **10**, 3430–3441 (2022).
3. Pang, H. *et al.* Novel Bionic Soy Protein-Based Adhesive with Excellent Prepressing Adhesion, Flame Retardancy, and Mildew Resistance. *ACS Appl. Mater. Interfaces* **13**, 38732–38744 (2021).
4. Kong, S. *et al.* Preparation of Soy Protein–Chitosan Adhesive Inspired by Marine Arthropod Shells with Superior Bonding Strength and Water Resistance. *ACS Sustainable Chem. Eng.* **13**, 20464–20475 (2025).
5. Pang, H. *et al.* Development of soy protein-based adhesive with high water resistance and bonding strength by waterborne epoxy crosslinking strategy. *International Journal of Adhesion and Adhesives* **100**, 102600 (2020).
6. Yao, J. *et al.* Cottonseed protein bioadhesive with high adhesion performance achieved by a synergistic dual-crosslinking strategy. *International Journal of Adhesion and Adhesives* **127**, 103514 (2023).
7. Zhang, Q. *et al.* Easy Preparation, High Water Resistance Glucose-Based Environment-Friendly Wood Adhesives. *ACS Sustainable Chem. Eng.* **12**, 7903–7912 (2024).
8. Hao, Z. *et al.* A fully bio-based soy protein wood adhesive modified by citric acid with high water tolerance. *International Journal of Biological Macromolecules* **253**, 127135 (2023).
9. Cesprini, E. *et al.* Bio-Based Tannin-Furanic-Silk Adhesives: Applications in Plywood and Chemical Cross-linking Mechanisms. *ACS Appl. Polym. Mater.* **5**, 4468–4476 (2023).
10. Zhang, X., Zhu, Y., Yu, Y. & Song, J. Improve Performance of Soy Flour-Based Adhesive with a Lignin-Based Resin. *Polymers* **9**, (2017).
11. Zheng, G., Pan, A., Xu, Y. & Zhang, X. Preparation of a superior soy protein adhesive with high solid content by enzymatic hydrolysis combined with cross-linking modification. *Industrial Crops and Products* **213**, 118446 (2024).
12. Li, D. *et al.* Chitosan used as a specific coupling agent to modify starch in preparation of adhesive film. *Journal of Cleaner Production* **277**, 123210 (2020).
13. Xi, X. *et al.* Environmentally friendly chitosan adhesives for plywood bonding. *International Journal of Adhesion and Adhesives* **112**, 103027 (2022).
14. LI, C. *et al.* Fully Biobased Adhesive from Glucose and Citric Acid for Plywood with High Performance. *ACS Appl. Mater. Interfaces* **14**, 23859–23867 (2022).
15. Peng, J., Liu, F., Feng, F., Feng, X. & Cui, J. Enhancing Environmentally Friendly Tannin Adhesive for Plywood through Hyperbranched Polyamide. *ACS Sustainable Chem. Eng.* **11**, 13805–13811 (2023).
16. Zuo, C. *et al.* A green one-pot strategy for sustainable fully lignin-based adhesives. *Green Chem.* **28**, 974–985 (2026).
17. Zhang, Y. *et al.* A High-Performance Bio-Adhesive Using Hyperbranched Aminated Soybean Polysaccharide and Bio-Based Epoxide. *Advanced Materials Interfaces* **7**, 2000148 (2020).
18. Pylypchuk, I. & Sipponen, M. H. Organic solvent-free production of colloidally stable spherical lignin nanoparticles at high mass concentrations. *Green Chem.* **24**, 8705–8715 (2022).
19. Moreno, A., Morsali, M. & Sipponen, M. H. Catalyst-Free Synthesis of Lignin Vitrimers with Tunable Mechanical Properties: Circular Polymers and Recoverable Adhesives. *ACS Appl. Mater. Interfaces* **13**, 57952–57961 (2021).
20. Abbadessa, A., Oinonen, P. & Henriksson, G. Characterization of Two Novel Bio-based Materials from Pulping Process Side Streams: Ecohelix and CleanFlow Black Lignin. *BioResources* **13**, 7606–7627 (2018).
